# Cutaneous lymphatics and skin diseases: mechanisms, imaging modalities, and future therapeutic strategies

**DOI:** 10.3389/fphys.2026.1754745

**Published:** 2026-05-05

**Authors:** Nuoran Chen, Duoduo Gu, Wanqing Yang, Xiaoqi Meng, Tingwei Liu, Dansheng Li, Yang Xu

**Affiliations:** 1 Department of Dermatology, The First Affiliated Hospital With Nanjing Medical University, Nanjing, China; 2 Department of Laser, Hospital for Skin Diseases, Institute of Dermatology, Chinese Academy of Medical Sciences & Peking Union Medical College, Nanjing, China; 3 Department of Dermatology, Beilun People’s Hospital, Ningbo, China

**Keywords:** cutaneous lymphatic vessels, imaging methods, lymphangiogenesis, skin diseases, therapeutic strategies

## Abstract

Cutaneous lymphatic vessels are essential for maintaining tissue homeostasis and coordinating immune defence, making them vital to skin health. Recent advances in molecular biology and immunology have revealed the association between lymphatic dysfunction and various dermatological conditions, including skin aging, psoriasis, systemic lupus erythematosus, and cutaneous squamous cell carcinoma. Lymphatic structure and function alterations influence immune regulation and play active roles in inflammatory responses and tissue repair processes. This study provides a systematic review of the biological characteristics of cutaneous lymphatic vessels, their mechanistic contributions to disease pathogenesis, and current lymphatic imaging methodologies. Emerging therapeutic strategies targeting lymphatic regulation represent a promising direction for dermatological interventions, with prospects for future research and clinical translation. By elucidating the pathophysiological mechanisms underlying cutaneous lymphatic activity, the aim of this review was to provide novel theoretical foundations and strategic insights for the prevention and treatment of related diseases.

## 1 Background

The cutaneous vascular system comprises blood and lymphatic vessel networks, with lymphatics playing a key role in maintaining tissue fluid homeostasis and mediating local immune responses. Previous investigations on lymphatic alterations in skin diseases are limited; however, recent advances in basic research on skin lymphatics and the development of advanced imaging techniques have revealed lymphatic alterations in conditions such as psoriasis, atopic dermatitis, rosacea, and skin cancers. This review provides a summary of the current insights into these findings.

## 2 Review

### 2.1 Structure and function of cutaneous lymphatic vessels

#### 2.1.1 Cutaneous lymphatic system

The cutaneous lymphatic system constitutes a structurally complex hierarchical network comprising initial lymphatic capillaries originating from the papillary dermis, subcutaneous pre-collecting, and collecting lymphatic vessels (Lund et al., 2016a; Oliver et al., 2020; Petrova and Koh, 2020). The initial lymphatic capillaries primarily drain and filter tissue fluid into the lymphatic system and facilitate the entry of antigen-presenting cells during immune responses (Melincovici et al., [[NoYear]]). Lymphatic fluid progresses from the initial capillaries through pre-collecting vessels into collecting vessels and is eventually transported to the lymph nodes and lymphatic trunks for integration into the systemic lymphatic circulation (Lund et al., 2016a). Unidirectional lymph flow is ensured by intraluminal valves within collecting vessels, with the functional segment between two adjacent valves termed the lymphangion (Swartz and Lund, 2012). The cutaneous lymphatic system transports tissue fluid, immune cells, macromolecules, antigens, pathogens, and lipids, thereby playing a crucial role in maintaining cutaneous fluid homeostasis and regulating immune responses (Lund et al., 2016a; Oliver et al., 2020; Petrova and Koh, 2020).

The role of cutaneous lymphatic vessels in immune responses is increasingly recognized. These vessels serve as conduits for antigen and immune cell transport and also actively modulate immune cell recruitment and activation through chemokines and adhesion molecules expressed by lymphatic endothelial cells. For instance, Langerhans cells contribute to shaping adult immune responses by promoting cutaneous lymphatic development, thereby indirectly modulating T cell responses in the lymph nodes (Sim et al., 2024). Furthermore, neutrophil recruitment and clearance by lymphatic vessels depend on the IL-17RC/CMTM4/NF-κB signalling axis, a mechanism particularly critical in inflamed or infected skin (Ni et al., 2024). Lymphatic dysfunction leads to the localised accumulation of inflammatory mediators, exacerbating inflammation, and promoting skin tumours and psoriasis-like dermatitis (Ni et al., 2024).

**Figure 1:**
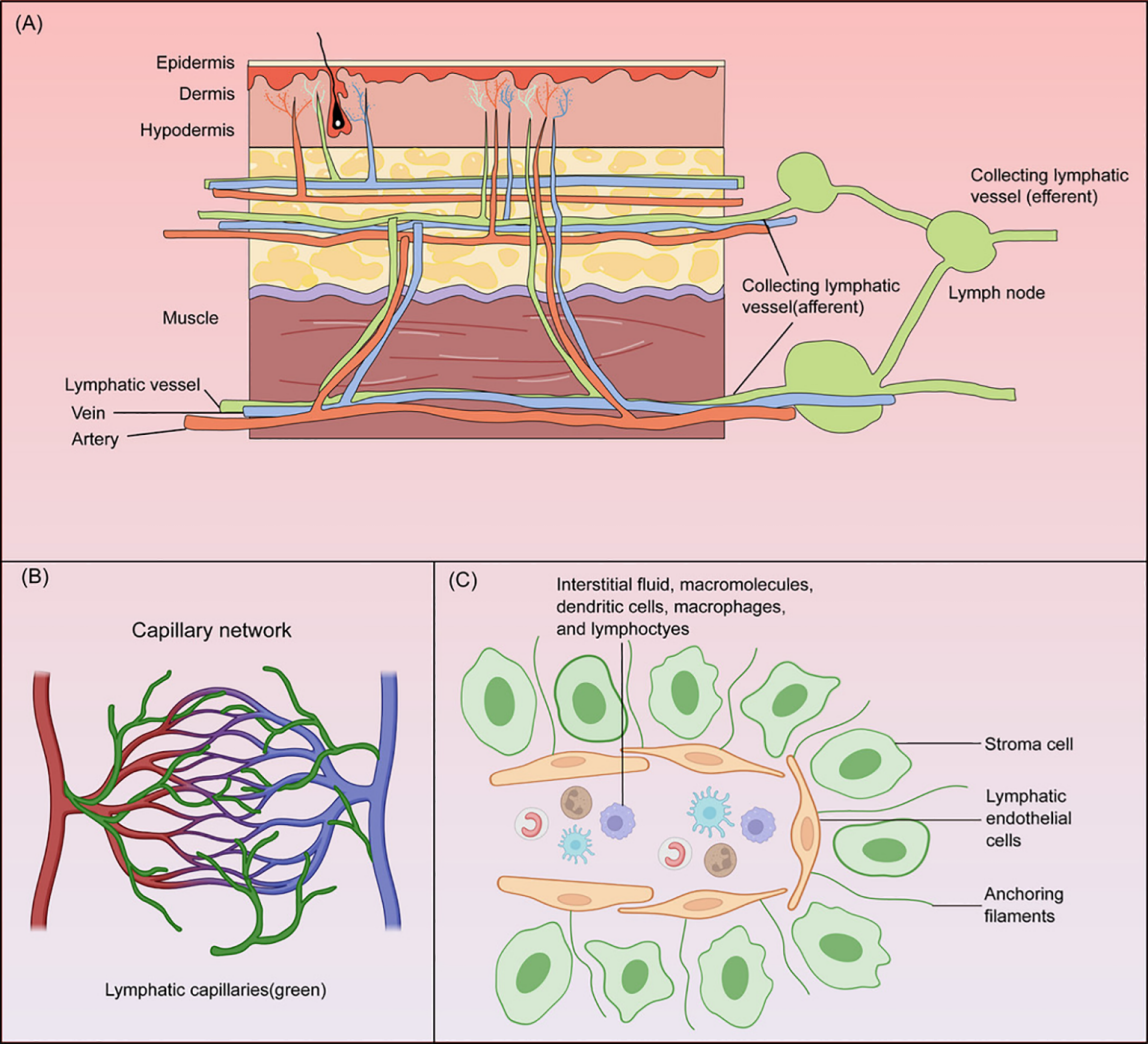
Structure of cutaneous lymphatic vessels. **(A)** Schematic diagram of skin and lymphatic system. **(B)** The lymphatic system (green) forms a reticulated network structure parallel to the vascular system (red and blue). **(C)** Lymphatic capillaries composed of single-layer loosely arranged endothelial cells facilitate the transport of dendritic cells, macrophages, and lymphocytes.

Regarding dermatological disease associations, multiple studies revealed significant connections between structural and functional lymphatic vessel abnormalities and various skin disorders. Abnormal lymphangiogenesis and structural remodelling of the vessel walls are the key pathological foundations of secondary lymphedema, which manifests as lymphatic dilation, smooth muscle cell hyperplasia, and transformation of junctional structures from button- to zipper-like configurations, ultimately leading to reduced fluid transport efficiency and exacerbated inflammation (Itai et al., 2024). Impaired cutaneous lymphatic function has been implicated in systemic lupus erythematosus, psoriasis, and cutaneous squamous cell carcinoma progression (Yang YL. et al., 2023; Itai et al., 2024). In patients with chronic spinal cord injury, the loss of neural control causes lymphatic dysfunction, characterized by reduced lymphatic dilation and enhanced lymphangiogenesis, contributing to impaired wound healing and increased infection susceptibility (Brunner et al., 2022). The structure of the lymphatic system is shown in **Figure 1**.

#### 2.1.2 Biology of lymphatic endothelial cells

Cutaneous lymphatic endothelial cells (LECs) are arranged in a flattened monolayer, interconnected by tight junctions. The current key molecular markers of LECs include prospero homeobox 1 (Prox1), vascular endothelial growth factor receptor-3 (VEGFR-3), podoplanin (PDPN), lymphatic vessel endothelial hyaluronan receptor-1 (LYVE1), and neuropilin-2 (NPN-2) (Lund et al., 2016a).

Prox1 is currently the most specific marker of human LECs (Truman et al., 2012). During the differentiation of LECs from venous endothelial cells, Prox1 expression precedes the acquisition of other lymphatic markers, including VEGFR-3, PDPN, and LYVE-1 (Kim et al., 2010). VEGFR-3, predominantly expressed in LECs (Hamrah et al., 2004), is essential for both embryonic development and adult lymphangiogenesis (Melincovici et al., [[NoYear]]; Shibuya, 2006). Vascular endothelial growth factor (VEGF)-C and -D bind to VEGFR-3 and, following proteolytic processing, can also activate VEGFR-2 to induce lymphatic and blood vessel formation (Hirakawa and Detmar, 2004). VEGFR-2 competitively inhibits VEGF-C binding to VEGFR-3, thereby suppressing LEC proliferation and lymphangiogenesis (Melincovici et al., [[NoYear]]; Albuquerque et al., 2009). Within the cutaneous vascular system, PDPN expression is restricted to LECs, and PDPN-deficient mice develop congenital lymphedema with impaired lymphatic formation and function (Proulx et al., 2013a). The PDPN-specific monoclonal antibody D2–40 is widely used for LEC identification (Kenney and Jain, 2008). LYVE-1, a CD44 homolog expressed in LECs and activated macrophages (Tunner et al., 1965), facilitates leukocytes and dendritic cell and their trafficking through the lymphatic system. NPN-2 enhances VEGFR-3 signalling through its interaction with VEGF-C (Melincovici et al., [[NoYear]]; Olsson et al., 2006). The expression levels of these markers vary across different lymphatic segments and predominantly express Prox1, PDPN, and LYVE-1; pre-collecting vessels mainly express Prox1 and LYVE-1, whereas collecting vessels primarily express Prox1 and PDPN (Wang et al., 2014; Yang and Oliver, 2014; Breslin et al., 2018).

### 2.2 Cutaneous lymphatic imaging techniques

In recent years, numerous novel lymphatic imaging techniques have emerged, providing valuable tools for investigating the pathophysiological roles of lymphatic vessels in the skin and other organs. Some of these methods are used in clinical practice, whereas others remain primary research tools.

Commonly used clinical imaging methods beyond conventional Doppler ultrasonography and magnetic resonance imaging include direct X-ray lymphangiography, magnetic resonance lymphangiography, lymphoscintigraphy, and fluorescence lymphangiography. However, most clinical techniques offer limited resolution for visualizing intradermal micro-lymphatic networks. In basic research, more advanced methodologies, such as photoacoustic imaging, optical coherence tomography, and two/multiphoton microscopy, are employed. When combined with fluorescent tracers or nanoprobes, these techniques allow detailed visualisation of the cutaneous lymphatic system. Nevertheless, the safety profiles of these contrast agents and nanoprobes in humans require further validation, currently limiting their application in animal studies. **Table 1** provides a summary of the fundamental principles, advantages, and limitations of these imaging techniques.

**Table 1:** Cutaneous lymphatic imaging techniques.

Technology	Main principle	Advantages	Disadvantages
Clinical Applications			
X-ray Lymphangiography (Zaleska and Olszewski, 2018; Laje et al., 2024)	Radiographic visualisation of lymphatic morphology using intradermal contrast agents (methylene blue/iodinated agents) via contrast drainage tracking	Enables visualisation of deep-seated lymphatic structures in the human body	Invasive procedure; technically challenging; requires high-dose contrast agent; risks include allergic reactions, pulmonary embolism, and contrast-induced nephropathy
High-frequency ultrasound (Tassenoy et al., 2016; Suehiro et al., 2017)	Image/data generation through ultrasound-tissue acoustic interaction	Non-invasive, convenient, relatively safe; suitable for infants and pregnant women	Limited resolution; poor visualisation of intradermal micro-lymphatics
Contrast-enhanced ultrasound (Jang et al., 2023)	Intradermal injection of sulphur hexafluoride lipid microspheres (Lumason/SonoVue) or other Food and Drug Administration–approved microbubbles followed by ultrasound scanning	Superior lymphatic visualisation compared to ultrasound; detects lymphatics not visible with indocyanine green (ICG)	Requires microbubble contrast injection; not widely adopted
Magnetic resonance imaging (Trinh et al., 2019; Yasunaga et al., 2021)	Signal generation from proton resonance under RF excitation in magnetic field	Non-invasive; radiation-free; three-dimensional (3D) reconstruction capability; visualisation of enlarged lymphatic trunks and abnormal lymph nodes	Long acquisition time; resolution insufficient for intradermal lymphatic network; contraindicated in patients with electrical, magnetic, or mechanical implants
Magnetic resonance lymphangiography (Mitsumori et al., 2016; Varagur et al., 2024)	Magnetic resonance visualisation of lymphatic system following interdigital injection of gadolinium-based contrast agents	Radiation-free; high-resolution imaging (detects single lymphatics ≥1 mm); slow contrast clearance enabling repeated examinations and real-time lymphatic flow monitoring	Fails to visualise intradermal micro-lymphatic network (<1mm vessels); contrast-related risks; patient compatibility restrictions
Lymphoscintigraphy (Chen et al., 2021)	Interdigital injection of ^9^^9^Tc^m^-labelled antimony sulphide (Tc-SbS) or dextran (^9^^9^Tc^m^-DX) tracers, followed by single-photon emission computerized tomography/computed tomography 3D imaging to track lymphatic transport of radiolabelled compounds	Minimally invasive procedure; technically straightforward; near-total body coverage with dynamic lymphatic drainage visualisation	Fails to visualise intradermal micro-lymphatics; contrast-related risks; low spatial resolution; radiation exposure concerns
Dye lymphography (Munn and Padera, 2014)	Subcutaneous dye injection (carmine/Prussian blue/Evans blue) tracks lymphatic drainage	Lymphatic pathway mapping	Limited to gross observation; potential false-positive staining; requires multiple tissue injections (invasive/time-consuming); historical use only
Near-infrared fluorescence imaging (Chen et al., 2021; Lauritzen et al., 2023)	Interdigital intradermal injection of ICG or sodium fluorescein, activated by near-infrared (780–900 nm) or visible light (300–760 nm), respectively, for lymphatic mapping	ICG fluorescence: real-time dynamic visualisation of lymphatic contraction with rapid imaging Sodium fluorescein: enhanced contrast differentiation between lymphatics and subcutaneous tissues	Fails to visualise intradermal micro-lymphatics; limited stability; slow imaging acquisition; inadequate depth penetration for deep lymphatics/nodes; contrast allergy risk (higher incidence with sodium fluorescein vs. ICG)
Novel *In Vivo* Research Technologies			
Photoacoustic Imaging (Forbrich et al., 2017; Kajita et al., 2020; Oh et al., 2022; Watanabe et al., 2022; Van Heumen et al., 2023)	Imaging via ultrasound signals generated by thermoelastic expansion in light-absorbing biological tissues	High-resolution non-invasive 3D lymphatic mapping; high optical contrast; cost-effective; real-time imaging	Limited penetration depth (enhanced with specialised array transducers); requires invasive contrast agent injection
Optical Coherence Tomography (OCT)/Optical Frequency Domain Imaging, (OFDI) (Banerjee et al., 2022)	Low-coherence light illumination enables 3D structural reconstruction of biological tissues through measurements of backscattered light time delays and interference patterns	Ultra-high resolution (micrometre-scale) visualisation of subcutaneous initial lymphatic microstructure; contrast agent-free repeated observations; OFDI-enabled dynamic monitoring	Limited imaging depth (<2 mm)
*In vivo* optical imaging (Proulx et al., 2013a; Proulx et al., 2013b)	Intradermal injection of polyethylene glycol-dye conjugates (e.g., P20D800) followed by *in vivo* fluorescence imaging under near-infrared illumination	Dynamic visualisation and quantification of cutaneous lymphatic drainage; reduced nodal retention vs. conventional ICG nanocolloids	High equipment costs; poor visualisation of intradermal lymphatic structures
Two/multi-photon imaging (Steven et al., 2011; Wang et al., 2021)	Simultaneous absorption of two/multiple photons, each contributing half the total energy required for fluorescence excitation	Simultaneous visualisation of immunofluorescence-labelled lymphatic structures and auto-fluorescent adjacent tissues (e.g., collagen fibres, blood vessels); enables high-resolution deep-tissue lymphatic imaging *in vivo* with nanoscale resolution	High equipment costs; requires fluorescent LYVE-1 monoclonal antibody injection
Immunohistochemistry of tissue sections (Wu et al., 2012)	Tissue section staining with podoplanin (PDPN) antibody	visualisation of the ultrastructural features in lymphatic capillaries	Limited to 2D structural analysis, lacks spatial context
Tissue block fluorescence staining (Wu et al., 2012)	PDPN antibody incubation followed by fluorescent secondary antibody labelling and confocal microscopy observation	Complete 3D visualisation of subepidermal lymphatic capillary networks	3D morphology limited to superficial lymphatics; pre-collecting vessels not observable

### 2.3 Cutaneous lymphatic vessels and skin diseases

Previous research on the structural and functional alterations of cutaneous lymphatic vessels in skin diseases is relatively limited. However, recent advances in lymphatic biology, imaging technologies, and functional assessment methodologies have facilitated substantial progress, providing novel insights into the pathophysiological mechanisms of these conditions.

#### 2.3.1 Psoriasis

Psoriasis is a chronic inflammatory skin disease characterized by epidermal hyperplasia, hyperkeratosis, immune cell infiltration, and vascular remodelling. Recently, studies have shown that the lymphatic system plays a significant role in tumour pathogenesis. Histological analyses of psoriatic lesions in patients and murine models reveal a significant increase in lymphatic vessel density and total lymphatic area, indicating active lymphangiogenesis. Specifically, immunohistochemical studies using PDPN as a lymphatic marker revealed significantly elevated lymphatic vessel counts and areas within psoriatic plaques compared with normal skin, along with a higher proliferation index (Ki-67), reflecting the active proliferation of LECS and keratinocytes (Detmar et al., 1994; Kunstfeld et al., 2004; Liew et al., 2012). In transgenic K-cyclin mouse models of psoriasis, severe lymphatic dysfunction has been observed, and this response is induced in the ear skin of 8-week-old female FVB wild-type mice. In imiquimod-induced psoriatic inflammation models, elevated inflammatory cytokine levels such as IL-17A, IL-22, and IL-23 activate STAT3 and NF-κB signalling pathways, resulting in exacerbated skin lesions (Boki et al., 2022).

Furthermore, lymphedema can trigger or exacerbate psoriasis. Kim et al., 2011 documented ipsilateral and unilateral psoriasis developing in lymphoedematous skin following unilateral mastectomy for breast cancer. Boki et al., 2022 demonstrated that K-cyclin transgenic (kCYC+/−) mice on an FVB/N background (8–12-week-old females) exhibiting severe lymphatic dysfunction developed more pronounced psoriatic-like inflammation following imiquimod induction. Cliff et al., 1999 observed increased lymphatic vessel density in the perilesional skin of patients with psoriasis but reduced lymphatic counts within plaques alongside impaired drainage function. Structural remodelling with lymphatic dilation influences psoriasis progression by facilitating immune cell migration and modulating the local immune microenvironment. A previous study revealed that the loss of EMILIN-1, an extracellular matrix (ECM) component, in fibroblasts derived from C57Bl/6 mice induces lymphatic dilation, disrupts tissue homeostasis, and promotes M1 macrophage polarisation, thereby aggravating psoriatic inflammation (Pivetta et al., 2022). Additionally, transcriptomic reprogramming of lymphatic and blood vascular endothelial cells from 4-mm punch biopsies of skin tissue from patients with psoriasis vulgaris alters cell adhesion molecules expression and ECM regulatory genes that sustain inflammatory cell infiltration and chronic inflammation (He et al., 2022). Consequently, lymphatic structural and functional alterations not only reflect psoriatic pathology but also actively support disease persistence and recurrence by creating a pro-inflammatory microenvironment.

Kunstfeld et al., 2004 demonstrated that a K14-VEGF-A transgenic mouse model of chronic skin inflammation exhibits a Th17-like disease phenotype similar to that of humans with psoriasis, establishing its relevance as an animal model of the disease. Their investigation revealed that lymphangiogenesis in this model, using 6-8-week-old mice of the FVB genetic background, primarily depends on VEGF-A/VEGFR-2 signalling, with a partial contribution from the VEGF-C/VEGFR-3 pathways (Huggenberger et al., 2010). LYVE-1 staining showed that systemic VEGFR-1 inhibition slightly increased the lymphatic vessel diameter, whereas blocking VEGFR-2 and VEGFR-3 significantly reduced the lymphatic density, with VEGFR-2 inhibition producing stronger effects. In oxazolone-challenged mice, a comparative analysis of VEGF-A+C or VEGF-A+D double-transgenic animals versus VEGF-A single-transgenic animals revealed significantly enhanced lymphatic network density, as determined by haematoxylin and eosin staining, accompanied by normalised ear thickness and complete resolution of inflammation. Localised administration of a VEGFR-3 inhibitor into the auricular skin increased lymphatic diameter and coverage while significantly reducing ear swelling (Kunstfeld et al., 2004). Another study showed that the intravenous administration of F8-VEGF-C in oxazolone-induced psoriatic dermatitis suppressed endogenous VEGF-C synthesis in mouse skin of 8-10-week-old female mice (Cousin et al., 2022), reduced lymphatic dilation and lymphedema, and alleviated psoriatic inflammation. These findings propose F8-VEGF-C as a potential therapeutic agent for psoriasis. Based on the aforementioned studies, it can be speculated that modulation of the VEGF/VEGFR signalling pathway may offer a promising therapeutic approach for psoriasis in the future.

#### 2.3.2 Atopic dermatitis

Atopic dermatitis (AD) is a chronic, relapsing, inflammatory skin disease characterized by a complex pathophysiology involving immune dysregulation and skin barrier impairment. Studies have confirmed increased dermal lymphatic vessel density and luminal dilation in patients with AD, representing a compensatory response to tissue fluid overload resulting from vascular hyperpermeability (Schwager and Detmar, 2019). However, the drainage function remains impaired despite lymphatic expansion (Savetsky et al., 2015).

Studies have revealed that impaired lymphatic transport capacity in mouse models with AD leads to localised accumulation of antigens and immune cells, prolonging and exacerbating inflammation, which are key mechanisms underlying the chronicity of AD (Zgraggen et al., 2013; Zhang et al., 2017). Conversely, AD-associated inflammation reciprocally alters lymphatic structure and function. Shi et al., 2012 used 12-week-old K14-IL-4 transgenic mice to demonstrate for the first time that AD-like inflammation induces dermal lymphangiogenesis. Immunostaining for PDPN, LYVE-1, CD11b, and VEGF-C in this model revealed significantly increased lymphatic density and diameter, with the upregulation of pro-lymphangiogenic factors (VEGF-C, VEGFR-3, ANG-1, and ANG-2) correlating with lesion severity.

#### 2.3.3 Rosacea

Studies, in which PDPN staining was employed, have shown abnormal lymphatic dilation across all subtypes of rosacea lesions, although no significant changes in lymphatic vessel density were observed (Schwab et al., 2011). Gomaa et al. reported significantly elevated VEGF, CD31, and D2–40 expression in lesional skin; however, D2–40 levels showed no correlation with disease duration, suggesting the potential involvement of lymphatic vessels in early stage pathogenesis (Gomaa et al., 2007). Patients with Morbihan’s disease, a variant of rosacea characterized by persistent facial oedema, develop solid facial swelling (Li et al., 2023). Furthermore, histological examination by Di Maria et al., 2024 revealed mild-to-moderate periorbital oedema, primarily involving the dermis with lymphatic dilation. Li et al. documented a case of Morbihan disease successfully managed with macro-focused high-intensity focused ultrasound, with *in vitro* experimental data suggesting that the therapeutic mechanism may involve enhanced cutaneous lymphatic drainage (Li et al., 2023).

A study by Duoduo Gu et al. revealed that the lesional skin of patients with rosacea exhibited downregulated gene expression of VEGFC, VEGFD, FLT4, LYVE1, and PDPN compared with those in healthy control skin. 6-8-week-old female BALB/c mice with chronic rosacea-like dermatitis induced by subcutaneous injection of LL-37 for 8 days showed significant inflammatory responses, dilation of dermal lymphatic vessels, and a reduction in lymphatic vessel number and density compared with those in the group treated with LL-37 for 48 h. *In vivo* small-animal imaging further indicated a significantly slowed lymphatic clearance. Long-pulsed Nd: YAG laser treatment ameliorated these alterations, which is consistent with clinical observations (Gu et al., 2025).

#### 2.3.4 Ultraviolet radiation-induced skin photodamage

Studies on chronically photodamaged facial skin in humans have also revealed a significant reduction in lymphatic vessels with localised oedema formation, indicating impaired lymphatic structure and function in ultraviolet-induced skin damage (Kajiya et al., 2006; Banerjee et al., 2022).

Chronic exposure of female hairless Skh1 mice(8 weeks old) to 40 mJ/cm ² UVB radiation three times weekly for 10 weeks induced photodamage manifestations such as erythema, significant lymphatic dilation, elevated permeability, aberrant leakage, and impaired drainage function. These pathological changes exacerbate oedema and inflammatory responses. Immunofluorescence staining of VEGF-A transgenic mice exposed to acute UVB radiation revealed that the upregulated VEGF-A expression enhanced lymphatic permeability, thereby aggravating oedema and inflammation. Conversely, VEGF-A signalling blockade ameliorated UVB-induced lymphatic dysfunction and other photodamage manifestations (Kajiya et al., 2006). Another study demonstrated that a single high-dose UVB exposure (200 mJ/cm²) led to the downregulation of VEGF-C expression in the dermis of 8-week-old female albino HR-1 hairless mice after 4 days (Kajiya et al., 2009). Collectively, these findings indicated that UVB-induced structural and functional impairments in cutaneous lymphatic vessels were mediated by VEGF-C suppression and VEGF-A upregulation. VEGF-C/VEGFR-3 signalling pathway activation and VEGF-A suppression may represent a potential therapeutic strategy for mitigating UVB-induced photodamage through enhanced lymphangiogenesis.

#### 2.3.5 Alopecia

Alopecia areata is an autoimmune dermatological disorder characterized by localised, non-cicatricial hair loss, primarily driven by peribulbar and intrafollicular lymphocytic infiltration. In contrast, androgenetic alopecia, which is predominantly influenced by hormonal factors and alterations in the follicular microenvironment, has not yet established a clearly defined relationship with lymphatic vessels.

Sundberg et al., 2016 used C3H/HeJ female mice at 10 weeks old as an alopecia areata model, and no alterations in cutaneous lymphatic vessel distribution or density were observed, but significant lymphatic dilation with localised oedema was identified. The degree of lymphatic dilation positively correlated with the expansion of alopecia patches. Peña-Jimenez et al., 2019 demonstrated that the cutaneous lymphatic system is an essential component of the hair follicle stem cell niche. Superficial lymphatic vessels maintain spatial polarity with hair follicles (consistently localised in the anterior aspect) and regulate hair cycle via Wnt signalling. In K15CrePR^+/T; Wls^Δ/Δ conditional knockout mice, lymphatic vessel dilation and enhanced drainage occurred during physiological hair follicle stem cell activation and pharmacologically-induced hair growth. Conversely, the specific ablation of lymphatic endothelial cells in Prox1CreERT2+/T; Rosa26-LSL-iDTR^KI/KI mice resulted in impaired hair growth.

Cutaneous lymphatic vessels influence hair growth through structural interconnection and signal transduction mechanisms. A contemporaneous study corroborated these findings, similarly identifying lymphatic vessels as a closely associated novel component of the hair follicle stem cell (HFSC) niche. During the telogen phase, lymphatic vessels maintain tight junctions with bulge stem cells. Disruption of the lymphatic network, either through pharmacological ablation or blockade of VEGFR3 signalling, triggers premature HFSC proliferation and accelerates the transition to the anagen phase. During physiological regeneration, HFSCs dynamically interact with lymphatic vessels; during early anagen (AnaII-III), lymphatic vessels temporarily dissociate from the stem cell niche and undergo dilation, resulting in reduced drainage capacity before reestablishing connections in the late anagen phase (AnaIV). HFSCs drive lymphatic remodelling through a ‘secretome switch’: telogen stem cells express Angiopoietin-like 7 (Angptl7) to promote lymphatic drainage, whereas activated stem cells switch to Angptl4 expression, triggering temporary lymphatic dissociation and reduced drainage. Disruption of this secretome switch (e.g., via sustained Angptl7 expression or Angptl7 knockdown) leads to either delayed hair cycling or asynchronous regeneration with follicular hyperplasia. Lymphatic dysfunction in Flt4^Chy^ mutant mice similarly causes asynchronous hair follicle regeneration, underscoring the importance of stem cell-driven lymphatic dynamics in coordinating tissue-wide stem cell behaviour (Gur-Cohen et al., 2019).

A previous study has identified high expression of Sostdc1 in LECs, with significantly elevated levels in the dorsal skin of 8-week-old female C57BL/6J mice during the anagen phase compared with those in the telogen phase. The underlying mechanism is mechanistically linked to Sostdc1-mediated activation of the Wnt pathway in dermal papilla cells (DPCs), further confirming that cutaneous lymphatic vessels promote hair follicle growth by secreting Sostdc1 to antagonize Bone Morphogenetic Protein signalling while activating the Wnt pathway in DPCs. This discovery identified Sostdc1 as a potential therapeutic target for alopecia treatment (Yoon and Detmar, 2022).

#### 2.3.6 Skin aging

Age-related dermatological conditions are becoming increasingly prevalent owing to homeostatic imbalance in the skin of the older adult population. Inflammation driven by cutaneous aging exacerbates local tissue damage and contributes to systemic inflammation, potentially accelerating organismal senescence. The lymphatic vessels play a pivotal role in maintaining fluid and immune homeostasis. In naturally aged human skin, lymphatic vessels exhibit reduced density, increased permeability, and impaired drainage. This elevated permeability compromises the capacity of the skin to mount effective immune responses and maintain interstitial fluid balance (Ryan, 2004; Yang Y. et al., 2023).

Karaman et al (Karaman et al., 2015)observed a significant reduction in lymphatic vessel density in the auricular skin of aged (18-month) C57BL/6 mice compared with young (2-month) and middle-aged (7-month) cohorts using a comparative age-group model. No lymphatic dilation was observed; however, aged lymphatic networks exhibited decreased branching complexity. Similarly, Kataru et al., 2022 demonstrated age-related impairment of the cutaneous lymphatic structure and function through immunofluorescence analysis in young (2–3 months) and aged (18–22 months) C57BL/6J mice. PDPN staining revealed lymphatic dilation in aged skin with downregulation of VEGFR3 in LECs, as confirmed by RT-PCR. Another proposed mechanism involves TGF-β/Activin signalling driving the endothelial-to-mesenchymal transition in aged cutaneous LECs, potentially mediated through YAP (Yes-associated protein)-induced suppression of VEGFR-3, Prox1, and LYVE1 (Yoshimatsu et al., 2020). Age-associated declines in lymphatic density and function correlate clinically with delayed wound healing and increased susceptibility to infection in older patients. For instance, the proportion of lymphatic vessels in upper eyelid tissues decreases significantly with advancing age, with better healing outcomes observed in lymphatically rich regions (Kashkouli et al., 2021; Nishioka et al., 2024). Furthermore, lymphatic calcification and remodelling have been identified as contributing factors to premature skin aging and refractory ulcer formation in genetic progeroid syndromes such as Werner syndrome, underscoring the pivotal role of lymphatic dysfunction in cutaneous aging (Ogata et al., 2021).

During aging, the VEGF-C/VEGFR-3 signalling pathway expression is downregulated, leading to impaired lymphatic remodelling and exacerbated dysfunction (Yang Y. et al., 2023). Furthermore, lymphatic endothelial cells exhibit increased apoptosis and senescence and diminished VEGFR-3 signalling, which further compromises lymphatic homeostasis. The reduced expression of VE-cadherin, a key endothelial junction protein, increases lymphatic permeability and promotes localised inflammation and oxidative stress, thereby creating a vicious cycle (Yang Y. et al., 2023). Studies indicate that lymphatic dysfunction accelerates skin aging and may also contribute to the pathogenesis of inflammatory dermatoses and tumour development (Ter-Ovanesyan et al., 2025).

#### 2.3.7 Pressure ulcer

A study investigating the effects of mechanical loading on cutaneous lymphatic function was conducted in eight healthy participants. Indocyanine green was injected intradermally into both forearms, and lymphatic drainage was monitored using a near-infrared fluorescence imaging system. A customized indenter was used to apply a uniaxial pressure of 60 mmHg to one arm for 45min as an experimental intervention. Images were acquired immediately after pressure release, following a 45-min recovery period. The analysis demonstrated that 60 mmHg of uniaxial pressure impaired dermal lymphatic formation and transport, characterized by reduced directional drainage and increased non-directional drainage. This lymphatic dysfunction may contribute to the pathogenesis of pressure-induced ulceration (Gray et al., 2016).

#### 2.3.8 Wound healing

Lymphangiogenesis plays a critical role in cutaneous wound healing, particularly in modulating inflammatory responses and maintaining tissue-fluid balance. Two proposed mechanisms regulate wound-associated lymphangiogenesis: the ‘self-organisation’ hypothesis, where LECs migrate along interstitial fluid flow to form networks autonomously, and the ‘sprouting’ hypothesis, wherein residual lymphatic vessels extend via endothelial budding (Jian et al., 2024). During wound repair, the inflammatory phase involves macrophages and neutrophils secreting VEGF-C/D and TNF-α to promote lymphangiogenesis, whereas TGF-β and IFN-γ may suppress this process (Corliss et al., 2016; Kataru et al., 2019; Singer, 2022; Willenborg et al., 2022). In the proliferative phase, lymphangiogenesis lags behind angiogenesis and is regulated by the VEGF-C/VEGFR-3 signalling axis and the transcription factor vascular endothelial zinc finger 1(VEZF1) (Gerald et al., 2013). During remodelling, nascent lymphatic vessels mature via collagen degradation, integrin-mediated adhesion, and hyaluronan-dependent mechanisms to establish functional networks. In chronic wounds, such as diabetic or venous ulcers, impaired VEGF-C expression compromises lymphangiogenesis, exacerbating tissue oedema and persistent inflammation (Amann-Vesti et al., 2004; Labanaris et al., 2009; Brunner et al., 2023). Targeted modulation of lymphangiogenesis represents a promising therapeutic strategy for chronic wounds. However, further studies are required to elucidate the optimal intervention timing and precise molecular mechanisms.

#### 2.3.9 Lymphedema

Secondary lymphedema, which frequently results from lymph node dissection or radiotherapy during cancer treatment, results in structural and functional abnormalities in the lymphatic system, subsequently inducing cutaneous fibrosis and increasing tissue stiffness. Lymphoscintigraphic classification studies comparing patients with secondary lower limb lymphedema to healthy controls revealed enhanced lymphangiogenesis during early-stage disease (types 1 and 2), characterized by VEGF-A/C-positive macrophage infiltration, Ki-67/Prox1-positive lymphatic endothelial cell proliferation, and increased lymphatic branching with reduced vessel diameter. Conversely, advanced lymphedema (types 3 and 4) exhibited structural remodelling of lymphatic capillaries featuring junctional transformation from ‘button-like’ to ‘zipper-like’ configurations, pericyte recruitment (SM22α+ cells), basement membrane deposition resembling collecting lymphatics, and functional impairment accompanied by collagen accumulation and downregulated LYVE-1 expression. The molecular mechanisms involved epidermal and macrophage-derived VEGF-A overexpression, coupled with reelin-mediated smooth muscle cell recruitment, promoting lymphatic maturation. These findings indicate that during lymphedema progression, cutaneous lymphatic capillaries undergo a transition from proliferative lymphangiogenesis to a dysfunctional state ‘zippering’, which may exacerbate fluid retention and infection risk. This pathophysiology supports stage-specific therapeutic strategies targeting inflammatory lymphangiogenesis in the early stages and intervening in junctional remodelling and smooth muscle activation in advanced cases (Itai et al., 2024).

#### 2.3.10 Systemic lupus erythematosus

Patients with systemic lupus erythematosus (SLE) exhibit a heightened sensitivity to ultraviolet radiation (UVR) and often develop intense cutaneous inflammatory responses, which can trigger systemic disease flares, including increased autoantibody production and organ damage. Studies utilizing skin biopsies from patients with SLE and two murine SLE models (6-15-week-old mice: magnetic resonance lymphangiography-Faslpr and imiquimod-induced SLE) have demonstrated lymphatic dilation and impaired lymphatic flow in human and murine SLE skin, with UVR-induced cutaneous swelling and enhanced infiltration of inflammatory cells, such as CD4+ T lymphocytes. Interventions restoring lymphatic flow—through manual lymphatic drainage (MLD) or transgenic approaches—alleviated UVR-induced cutaneous inflammation in lupus-prone mice, reducing ear swelling, CD4+ T cell infiltration, and IFN-γ expression. These interventions also suppress germinal centre B cells, plasmablasts, and CD4+ T cell responses in draining lymph nodes. Long-term MLD treatment reduced splenomegaly and decreased titres of multiple autoantibodies (e.g., anti-histone 2B, anti-β2-glycoprotein). Mechanistically, improved lymphatic flow upregulates CCL21 expression in lymph node fibroblastic reticular cells and enhances reactive oxygen species production by Ly6C hi monocytes, thereby limiting plasmablast expansion. These findings establish lymphatic dysfunction as a critical factor underlying photosensitivity and aberrant B cell responses in SLE. Restoration of lymphatic flow may represent a novel therapeutic strategy for mitigating cutaneous and systemic autoimmunity in SLE (Howlader et al., 2025).

Immunohistochemical analysis using D2-40, a specific marker for lymphatic endothelial cells, revealed abnormal lymphatic vessel density and distribution in renal and cutaneous tissues of patients with SLE and murine models(male Balb/c mice, 7–8 weeks old), indicating a significant correlation between lymphangiogenesis and inflammatory activity in the disease (Wang et al., 2025). Excessive lymphangiogenesis is associated with aggravated glomerular and interstitial inflammation. VEGFR-3 signalling pathway activation promotes lymphatic expansion and modulates the local immune system. The VEGFR-3 inhibitor, SAR131675, demonstrated therapeutic potential by suppressing renal lymphangiogenesis, attenuating kidney inflammation, and mitigating histological damage, thus highlighting the pathogenic role of lymphatic vessels in lupus nephritis (Wang et al., 2025). Therefore, structural and functional alterations in lymphatic networks represent a foundation for localised tissue damage and a central mechanism in systemic autoimmunity. Mechanistically, SAR131675 ameliorates murine lupus nephritis by inhibiting the TLR7/MyD88/IFN-α signalling axis and reducing pro-inflammatory cytokine/chemokine expression, suggesting that targeted lymphangiogenesis regulation may offer a novel strategic approach for controlling SLE-related inflammation and autoimmune responses (Wang et al., 2025).

#### 2.3.11 Systemic sclerosis

Systemic sclerosis (SSc) is an autoimmune disorder characterized by vascular abnormalities, immune dysregulation, and progressive fibrosis. Cutaneous lymphatic vessels evaluation in patients with SSc revealed a significant reduction in dermal lymphatic density compared with healthy controls. Specifically, lymphatic vessels are markedly diminished in the papillary and reticular dermis of patients with SSc. In early stage SSc, no significant difference in papillary dermal lymphatic density was observed relative to the controls, although a decreasing trend was noted in the reticular dermis. In contrast, advanced SSc exhibited significantly reduced lymphatic counts in both dermal layers, with the papillary layer showing a more pronounced loss than in early-stage disease. These findings suggested that lymphatic depletion was initiated in the reticular dermis and progressively extended to the papillary layer. This study indicates that lymphatic microangiopathy correlates with the progression of cutaneous involvement in SSc, and that the progressive loss of lymphatic vessels may play a pivotal role in the transition from early oedematous phases to overt fibrotic manifestations of the disease (Manetti et al., 2011).

A comparative study of forearm skin biopsy specimens from nine patients with SSc and seven age-matched healthy controls revealed significantly reduced lymphatic density in the reticular dermis of patients with SSc, with a similar trend observed in the papillary dermis. Patients with SSc exhibit increased luminal areas of lymphatic vessels (suggesting compensatory dilation), particularly in the peri-glandular lymphatics. Notably, peri-glandular lymphatic density remained preserved in patients with SSc compared with controls, which is potentially attributable to local VEGF-C secretion from the glandular tissue. The proportion of non-lumenized vascular profiles was significantly decreased in patients with SSc; however, the total vascular count and area showed no significant differences. These findings indicate that cutaneous SSc pathology is characterized by selective lymphatic reduction with relative preservation of the peri-glandular lymphatics. The combined phenomenon of lymphatic depletion and dilation may disrupt interstitial fluid balance and impair lymphatic drainage, contributing to disease progression and clinical manifestations. This insight provides a novel perspective for understanding the pathogenesis (Rossi et al., 2010).

A separate study identified the lymphatic endothelial-to-mesenchymal transition (Ly-EndMT) as a potential novel mechanism linking lymphatic dysfunction to cutaneous fibrosis in SSc. Researchers observed that 40% of lymphatic vessels in the skin of a patient with SSc contained transitional cells co-expressing LYVE-1 and α-SMA, a phenomenon absent in healthy skin. The serum of the patient with SSc and TGF-β1 stimulation induced morphological transition in human dermal lymphatic microvascular endothelial cells from polygonal to spindle-shaped, with downregulation of Prox1/LYVE-1 and upregulation of α-SMA/S100A4/COL1A1/Snail1, along with enhanced contractile capacity. This transition appears mediated by elevated TGF-β1 levels in SSc serum, revealing a potential pathway through which Ly-EndMT connects lymphatic impairment with fibrotic progression in SSc (Rosa et al., 2023).

#### 2.3.12 Cutaneous squamous cell carcinoma

A study investigating the dynamic remodelling of the lymphatic-centred immune microenvironment during UVR-induced cutaneous squamous cell carcinoma (cSCC) pathogenesis revealed stage-specific alterations in murine models (female hairless SKH-1 mice, 12 weeks old). Early UV-damaged skin exhibited reduced lymphatic vessel density with increased CD4+ T cell infiltration and decreased CD8+ T-cell numbers. As the lesions progressed to invasive cSCC, the lymphatic density significantly increased, with further exacerbation of CD4+ and CD8+ T cell imbalances. YAP1 expression mirrored lymphatic changes, being lowest in photoaged skin and significantly elevated in cSCC, whereas Piezo1 demonstrated an inverse pattern, peaking during photoaging before a gradual decline. In human head and neck SCC, VEGFC overexpression is positively correlated with YAP1 levels. Notably, the combination of high lymphatic density and CD8+ T-cell infiltration predicted improved patient survival. These findings establish that the lymphatic-centred immune microenvironment undergoes adaptive remodelling during cSCC development via YAP1/VEGFC- and Piezo1-mediated mechanisms. Dynamic changes in YAP1/Piezo1 expression, lymphatic architecture, and immune cell profiles may enable early detection and intervention during cSCC progression (Yang YL. et al., 2023).

The immune microenvironment of cSCC constitutes a dynamic and paradoxical ecosystem characterized by coexisting pro-tumour and anti-tumour forces. Key features include increased immunosuppressive cells (e.g., Tregs and MDSCs), impaired antigen presentation, macrophage polarisation toward the M2 phenotype, metastasis-promoting lymphangiogenesis, and a complex network of pro-tumour cytokines (Saeidi et al., 2023). cSCC exhibits elevated lymphatic vessel density and relative lymphatic area (Sedivy et al., 2003; Miyahara et al., 2007), with VEGF-C overexpression serving as a critical mediator of lymphangiogenesis. PDPN is overexpressed in cSCC and is positively correlated with tumour differentiation grade, invasion depth, and lymph node metastasis risk, making it a potential prognostic biomarker (Moussai et al., 2011; Kim et al., 2015; Wojciechowska-Zdrojowy et al., 2016; Arimoto et al., 2018).

Furthermore, cSCC cases exhibiting lymphovascular invasion (LVI) demonstrate a significantly elevated risk of lymph node metastasis (Moore et al., 2005) and disease-specific mortality (Carter et al., 2013). LVI represents an independent high-risk factor for cSCC and should be managed as urgently as neurotropic invasion. Future studies are warranted to elucidate the causal role of LVI in tumour progression (Ryan et al., 2024).

#### 2.3.13 Basal cell carcinoma

Basal cell carcinoma (BCC) typically remains localised with an exceptionally low metastatic rate; however, its local invasiveness and recurrence significantly contribute to patient morbidity (Lang et al., 2024). Lymphatic vessels play crucial roles within the tumour and participate in tumour growth, metastasis, and immune regulation. Studies have demonstrated significant alterations in lymphatic vessel density and related molecular expression within BCC tumour tissues and the surrounding stroma, suggesting that lymphangiogenesis may contribute to BCC development and progression.

SOX18, a key transcription factor involved in vascular endothelial cell differentiation, regulates angiogenesis and lymphangiogenesis. Its expression is significantly upregulated in non-melanoma skin cancers, including BCC and SCC, with notably higher levels observed in high-risk BCC subtypes than in low-risk variants. The positive correlation between SOX18 expression and tumour stromal vascular density further supports its potential role in promoting tumour evolution and progression by enhancing vascular and lymphatic formation (Neinaa et al., 2020). Furthermore, differential expression of the lymphangiogenesis-related protein PDPN in BCC correlated with increased tumour aggressiveness (Jabour et al., 2022).

#### 2.3.14 Cutaneous melanoma

Cutaneous melanoma is a highly aggressive malignancy characterized by early lymph node metastasis and a poor prognosis. Lymph node metastasis plays a crucial role in melanoma staging, prognosis, and treatment strategies; however, the cutaneous lymphatic network is closely associated with melanoma development and metastasis. Studies have demonstrated that lymphatic micro-vessel density (LMVD), particularly peritumoral LMVD, significantly correlates with lymph node metastasis and patient survival (Emmett et al., 2010; Pastushenko et al., 2015). Moreover, VEGF-C and VEGF-D are highly expressed in metastatic melanoma and are associated with unfavourable outcomes (Boone et al., 2008; Špirić et al., 2015). The underlying mechanism may involve tumour and inflammatory cells that promote lymphangiogenesis through the secretion of growth factors (e.g., EGF and PDGF-C) and chemokines (e.g., CCL21) (Lee et al., 2011; Takekoshi et al., 2012).

Studies have shown that lymphangiogenesis is significantly enhanced in the melanoma microenvironment, closely correlating with tumour invasiveness and metastatic potential. Markers, including LYVE-1, PDPN, and VEGFR-3, show elevated expression in melanoma-invaded lymph nodes, indicating active lymphatic remodelling (Reger de Moura et al., 2021). The lymphangiogenic protein CD147 promotes melanoma-associated lymphangiogenesis and metastasis *in vivo* by regulating the transcription factor PROX-1, highlighting its potential as a therapeutic target (Reger de Moura et al., 2021). Furthermore, multifunctional optical coherence tomography tracking in murine melanoma models revealed that lymphangiogenesis precedes angiogenesis, with lymphatic dilation serving as an early indicator of tumour invasion (Lai et al., 2021). The interaction between lymphatic vessels and immune cells within the tumour microenvironment further influences immune escape and tumour progression. Lymphatic endothelial cells modulate antitumor immune responses, potentially facilitating immune evasion, while simultaneously representing promising targets for immunotherapy (Femel et al., 2024). Analysis of mice lacking dermal lymphatic vessels (K14-VEGFR3-Ig transgenic mice, 10-12-week-old males) and human metastatic cutaneous melanoma samples from the TCGA database demonstrated a positive correlation between lymphatic signature scores and immune markers (e.g., CD45, CD8A, CD4, and FOXP3) and inflammatory cytokine expression. Tumour growth remained unchanged in K14-VEGFR3-Ig mice; however, cytokine expression and leukocyte infiltration were significantly reduced, accompanied by impaired lymphatic drainage and diminished dendritic cell transport to the draining lymph nodes. These mice also exhibited reduced immune responses to intradermal vaccination, characterized by reduced antigen-specific CD8+ T cells and IFN-γ production. Conversely, adoptively transferred cytotoxic T cells demonstrated enhanced tumour control in K14-VEGFR3-Ig mice, with increased intra-tumoural T-cell infiltration and IFN-γ production. Spontaneous lung metastasis reduced in these mice; however, pulmonary colonisation following intravenous tumour cell injection remained unchanged, suggesting that metastatic suppression was linked to alterations in the primary tumour microenvironment. Collectively, these findings highlight the critical role of lymphatic remodelling and drainage in establishing tumour-associated inflammation and immunity and position lymphatic vessels as prognostic biomarkers and therapeutic targets in melanoma immunotherapy (Lund et al., 2016b).

Using a similar model, other researchers found that lymphatic vessels play an important role in the adipose tissue of melanoma patients. In a melanoma model using C57BL/6, male, 10–11-week-old K14-VEGFR3-Ig transgenic mice, they observed marked atrophy of peritumoral adipose tissue and a reduction in adipocyte size. The number of CD301^+^ cells was significantly increased in the peritumoral adipose tissue, and melanoma cells implanted in the fat pad exhibited accelerated tumour growth and enhanced vascularisation. In the human melanoma TCGA database, high expression of the adipocyte marker FABP4 was correlated with shorter overall survival of patients. Moreover, FABP4 expression was positively correlated with the M2 macrophage marker CLEC10A (CD301) and IL-6 expression. These findings suggest that lymphatic insufficiency promotes the accumulation of M2-type tumour-associated macrophages in adipose tissue, thereby accelerating melanoma growth (Wagner et al., 2019).

Steinskog et al., 2016 found that following severe impairment of dermal lymphangiogenesis, orthotopic transplantation of KHT-1 sarcoma in Chy mice (C3H background) led to significantly accelerated tumour growth. The rate of lymphatic drainage from the peritumoral skin was markedly lower in Chy mice compared to wild-type mice. Total macrophage count in the peritumoral area was reduced in Chy mice, whereas the proportion of M2 macrophages remained unchanged, resulting in an elevated M2/M1 ratio. Under normal conditions, lymphatic vessels exert an anti-tumor suppressive role by facilitating drainage and maintaining M1 macrophage infiltration. Lymphatic dysfunction leads to reduced immune surveillance, a shift toward M2 polarisation, and increased IL-6 levels, thereby accelerating tumour growth. The researchers hypothesize that restoring lymphatic function may enhance anti-tumor immunity. Fankhauser et al., 2017 found that in B16 melanomas overexpressing VEGF-C, lymphatic endothelial cells (LECs) highly expressed CCL21. Through CCR7, CCL21 recruited naïve T cells (CD44^-^CD62L^+^) into the tumor microenvironment. Following immunotherapy-induced tumour cell death, these naïve T cells were activated by local antigens and danger signals, generating effector and memory T cells and leading to antigen spreading. Blockade of VEGFR-3 (inhibiting lymphangiogenesis) reduced the efficacy of immunotherapy, whereas melanomas with high VEGF-C expression (active lymphangiogenesis) were more sensitive to immunotherapy. VEGFR-3 blockade using an αR3 antibody suppressed lymphangiogenesis in B16-OVA/VC tumours and significantly diminished the response to immunotherapy. Although lymphangiogenesis may promote immunosuppression in the absence of immunotherapy, in the presence of immunotherapy it significantly enhances therapeutic efficacy by recruiting naïve T cells as a ‘reservoir’. Under different therapeutic strategies, lymphatic vessels play distinct roles.

#### 2.3.15 Cutaneous T-cell lymphoma

Cutaneous T-cell Lymphoma (CTCL) is a group of non-Hodgkin T-cell lymphomas that primarily affects the skin and exhibits heterogeneous clinical presentations. Common subtypes include mycosis fungoides and Sézary syndrome.

The cutaneous lymphatic system undergoes significant alterations during CTCL progression. Pathological lymphatic dilation and neogenesis in CTCL are closely associated with tumour-related inflammatory responses and cytokine secretion, particularly the upregulation of pro-lymphangiogenic factors, such as VEGF-C. Elevated VEGF-C expression promotes lymphangiogenesis and facilitates malignant cell dissemination to the lymphatic system (Dummer et al., 2021). Furthermore, the intraluminal accumulation of tumour cells within lymphatic vessels compromises lymphatic function, impairs local immune surveillance, and promotes tumour immune escape. In CTCL skin lesions, the bidirectional interaction between lymphatic vessels and tumour cells establishes a pathological cycle that exacerbates local symptoms (e.g., as erythema, plaques, and tumour formation) and also creates pathways for distant metastasis (Arora and Gogia, 2020).

#### 2.3.16 Primary Cutaneous B-cell lymphoma

Cutaneous lymphomas are non-Hodgkin lymphomas that do not involve the lymph nodes or other non-cutaneous organs at the time of diagnosis. Based on current national guidelines, they are classified as T-cell lymphoma, B-cell lymphoma, or other entities (Dippel et al., 2018).

Accounting for approximately 22% of cutaneous lymphomas, CBCL includes primary cutaneous follicular centre lymphoma, primary cutaneous marginal zone lymphoma, and primary cutaneous diffuse large B-cell lymphoma. A retrospective analysis of biopsy specimens from 30 patients with CBCL, evaluated by haematoxylin and eosin staining and immunohistochemistry for CD20 and D2-40, demonstrated extensive lymphatic invasion across all three subtypes. The authors concluded that lymphatic invasion is relatively common in primary CBCL, although no significant association with the recurrence rate was identified in this cohort (Bertlich et al., 2023).

### 2.4 Therapeutic strategies

No clinical studies have validated the efficacy of lymphatically targeted pharmacological or cellular therapies in humans. However, numerous preclinical studies in animal models have provided substantial foundational data and theoretical support for future clinical applications.

Increasing evidence supports the therapeutic potential of modulating lymphatic drainage in disease management. In a murine model of inflammatory arthritis, ginsenoside Rg1 demonstrated anti-inflammatory efficacy by enhancing lymphatic drainage, reducing joint inflammation and bone erosion, and improving clinical symptoms in TNF-α transgenic mice, highlighting its potential as a lymphatic-modulating agent (Hou et al., 2020). Transplantation of adipose-derived stem cells in a radiation-induced lymphedema model promotes lymphatic endothelial cell proliferation and ameliorates cutaneous fibrosis and lymphatic dilation, potentially through intussusceptive lymphangiogenesis, thereby effectively alleviating lymphedema (Ogino et al., 2020). In a secondary lymphedema model, the neuropeptide substance P reduced lymphedema and inflammation by promoting M2 macrophage polarisation and inhibiting the NF-κB/NLRP3 signalling pathway, suggesting its immunomodulatory therapeutic potential (Zhou et al., 2023).

Current research on promoting cutaneous lymphangiogenesis has primarily focused on the VEGFC/VEGFR-3 and CGRP/p44/42 MAPK signalling pathways. Studies have demonstrated that the F8-VEGF-C fusion protein expands functional lymphatic vessels, enhances lymphatic drainage, and reduces infiltration of CD4+ and γδ T-cells in two distinct psoriasiform mouse models, thereby effectively ameliorating chronic skin inflammation (Schwager et al., 2018). 5-aminolevulinic acid photodynamic therapy and artichoke polyphenols improve lymphatic function by upregulating the VEGFC/VEGFR-3 signalling pathway and enhancing intercellular junctions (e.g., VE-cadherin) in LECs, thereby exerting anti-aging effects (D’Antuono et al., 2018; Yang et al., 2022). Mesenchymal stem cells promote lymphangiogenesis via the paracrine VEGF-C secretion, accelerating wound healing in diabetic mice (Conrad et al., 2009; Robering et al., 2018; Fuchs et al., 2023). Moreover, regulatory factors, such as lncRNA-GAS5, can upregulate VEGFR-3 expression in LECs (He et al., 2019; Rezvanian et al., 2021) and improve lymphatic function under hyperglycaemic conditions. Additionally, calcitonin gene-related peptide and adrenomedullin-2/intermedin directly activate p44/42 MAPK phosphorylation in cutaneous lymphatic endothelial cells, thereby modulating their biological functions (Hasan et al., 2024).

## 3 Conclusion

The cutaneous lymphatic system, essential for maintaining skin homeostasis and immune defence, is increasingly recognized as a key factor in the pathogenesis of various dermatological disorders. Current research suggests that future investigations in this field are likely to focus on the following. First, elucidating the molecular mechanisms by which cutaneous lymphatic vessels regulate skin immunity remains a priority. Secondly, quantitative and dynamic analyses are required to clarify how lymphatic morphological and functional alterations relate with disease progression. Finally, targeted therapeutic strategies to address lymphatic dysfunction requires further systematic exploration. Advancements in these areas will provide a theoretical and technical foundation for the precision medicine of lymphatic-related cutaneous diseases.
